# Interventions in Nicotinamide Adenine Dinucleotide Metabolism, the Intestinal Microbiota and Microcin Peptide Antimicrobials

**DOI:** 10.3389/fmolb.2022.861603

**Published:** 2022-03-14

**Authors:** Fernando Baquero, Rosa del Campo, José-Luis Martínez

**Affiliations:** ^1^ Department of Microbiology, Ramón y Cajal University Hospital and Ramón y Cajal Institute for Health Research (IRYCIS), Madrid, Spain; ^2^ Network Center for Research in Epidemiology and Public Health (CIBER-ESP), Madrid, Spain; ^3^ Network Center for Research in Infectious Diseases (CIBER-INFEC), Madrid, Spain; ^4^ National Center for Biotechnology (CNB-CSIC), Madrid, Spain

**Keywords:** NAD metabolism, microbiota, NAD+ enhancers, NAD+ inhibitors, microcins, antibiotic persisters, antibiotics, fecal microbiota transplantation

## Abstract

A proper NADH/NAD + balance allows for the flow of metabolic and catabolic activities determining cellular growth. In *Escherichia coli*, more than 80 NAD + dependent enzymes are involved in all major metabolic pathways, including the post-transcriptional build-up of thiazole and oxazole rings from small linear peptides, which is a critical step for the antibiotic activity of some microcins. In recent years, NAD metabolism boosting drugs have been explored, mostly precursors of NAD + synthesis in human cells, with beneficial effects on the aging process and in preventing oncological and neurological diseases. These compounds also enhance NAD + metabolism in the human microbiota, which contributes to these beneficial effects. On the other hand, inhibition of NAD + metabolism has been proposed as a therapeutic approach to reduce the growth and propagation of tumor cells and mitigating inflammatory bowel diseases; in this case, the activity of the microbiota might mitigate therapeutic efficacy. Antibiotics, which reduce the effect of microbiota, should synergize with NAD + metabolism inhibitors, but these drugs might increase the proportion of antibiotic persistent populations. Conversely, antibiotics might have a stronger killing effect on bacteria with active NAD + production and reduce the cooperation of NAD + producing bacteria with tumoral cells. The use of NADH/NAD + modulators should take into consideration the use of antibiotics and the population structure of the microbiota.

## Introduction

Bacterial growth rates depend on the energetic quality of available products in the environment, the bacterial chemosphere (Baquero et al., 2019), and the bacteria’s ability of bacteria to adjust their metabolism to nutrients availability, while efficiently maintaining metabolic homeostasis. During fermentation, for instance, reduced nicotinamide adenine dinucleotide dehydrogenase (NADH) are responsible for carrying high-energy electrons involved in the oxidative recycling of the tricarboxylic cycle product NADH back to the oxidized form of nicotinamide adenine dinucleotide NAD+. NAD+ is required to reconstitute NADH; consequently, a proper NADH/NAD + balance allows for the flow of metabolic and catabolic activities determining bacterial cell growth. NAD+ is a key regulator of gene expression, and can be used as substrate and cofactor for reactions ensuring genomic stability, as well as for the expression of NAD + -dependent enzymes. In *Escherichia coli*, more than 80 NAD + dependent enzymes are involved in all major metabolic pathways, a paradigmatic example being the NAD + dependent deacetylases, also known as sirtuins, critical components of bacterial metabolism and stress control (Hentchel and Escalante-Semerena, 2015; Bheda et al., 2016). NAD+ is required in the post-transcriptional modifications of peptides and proteins (Xie et al., 2020).

In most bacterial cells NAD+ is synthesized through a *de novo* synthesis pathway, starting from tryptophan, nicotinic acid, or niacin, and salvage pathways I and/or II, using nicotinamide molecules. The regulation of bacterial NAD + biogenesis depends on the sensing of global carbon-nitrogen levels (Santos et al., 2020). In fact, the suppression of enzyme NadE, involved in the last catalyzing last of NAD biosynthesis, NadE, has been shown to result in a loss of bacterial viability (Rodionova et al., 2014). In humans NAD + homeostasis is critical for maintaining health; for instance delaying the aging process, and preventing oncological and neurological diseases (Braidy and Liu, 2020; Ahmed and Braidy, 2021; Villalva et al., 2021). Conversely, NAD biogenesis also contributes to the pathogenesis of tumoral and inflammatory diseases (Katsyuba et al., 2020).

## NAD Metabolism: The Role of Intestinal Microbiota

**FIGURE 1:**
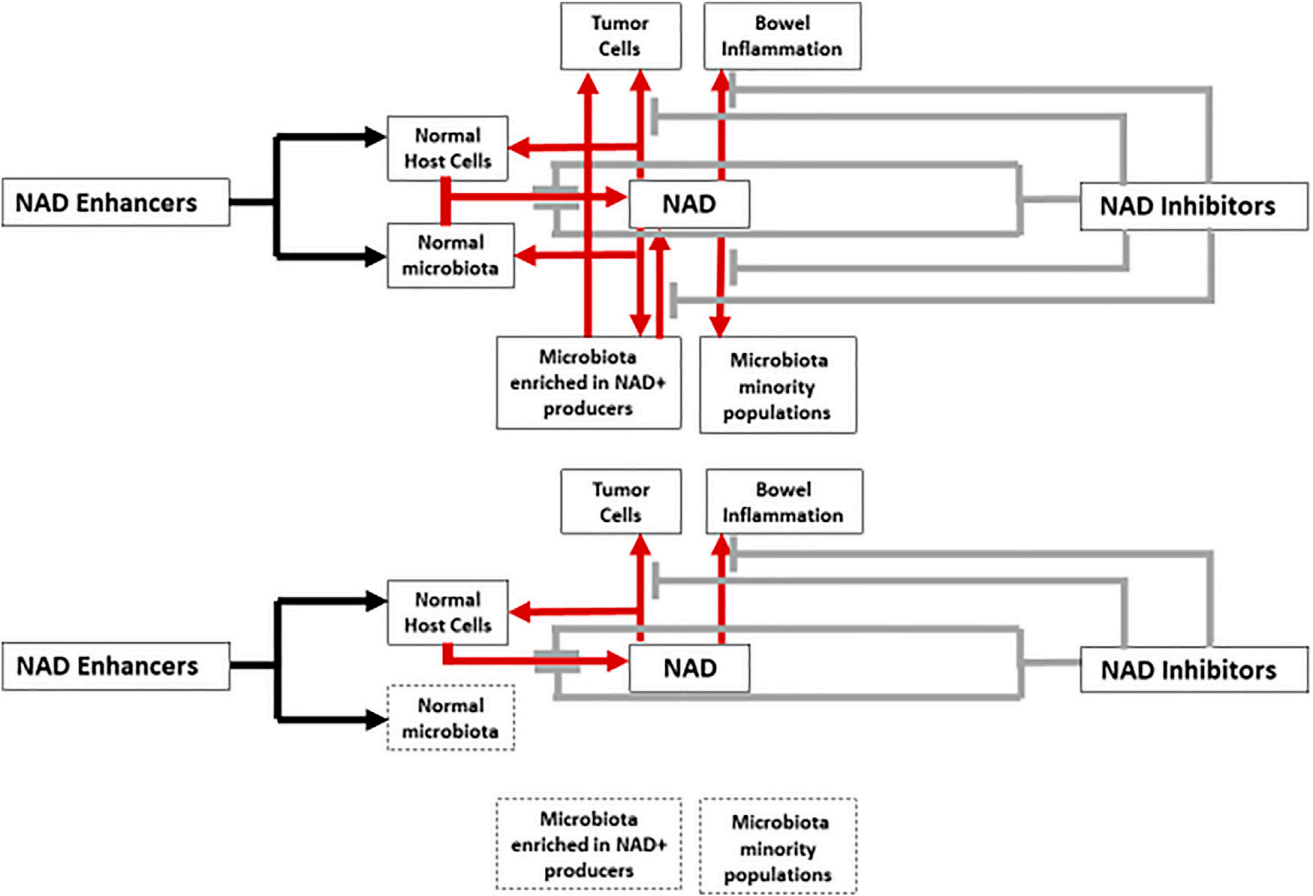
Interactions between NAD metabolism enhancers, inhibitors, host cells and microbiota. Red arrows and grey brakes correspond to the increasing (boosting compounds) or decreasing (inhibitory compounds) effects on NAD levels, respectively. In the lower part of a figure the same schema is reproduced with the expected inhibitory effect of antibiotic therapy, reducing or eliminating the contribution of the microbiota on NAD metabolism (spotted boxes).

NAD is an universal coenzyme, present in all types of cells, including bacterial. Consequently, NAD biosynthesis in humans and animals depends on their eukaryotic cells and prokaryotic cells of the intestinal microbiota, acting as a genuine organ in mammals (Baquero and Nombela, 2012). (Figure 1).

## Microbiota and Enhancers of NAD Metabolism

There is evidence indicating that NAD + plays important roles not only in mitochondrial functions and energy metabolism, but also in inflammation, calcium homeostasis, and most importantly mitigating numerous neurological diseases, including ischemic brain damage, Alzheimer’s disease, and Parkinson’s disease. Elevated NAD levels also exert a protective effect against oncogenesis (Ma et al., 2012). In addition, enhancers of NAD biogenesis can compensate for decreased cellular NAD levels in aging (Imai and Guarente, 2014; Fang et al., 2017; Covarrubias et al., 2021). Mammalian cells cannot import NAD+; so that commercial oral NAD-boosting supplements have been developed, using exogenous NAD^+^ precursors, such as L-tryptophane, nicotinic acid, nicotinamide-mononucleotide, nicotinamide-riboside, and dihydronicotinamide-riboside (Ratajczak et al., 2016; Sinclair, 2018; Reiten et al., 2021; Zapata-Pérez et al., 2021). The use of such boosting compounds is accepted by the European Food Safety Authority Panel (Baquero et al., 2021). NAD-production can also be achieved with activators of nicotinamide phosphoribosyltransferase (NAMPT), a key enzyme in the *ex-novo* production of NAD (Hua et al., 2021). Another possibility for maintaining appropriately high NAD levels is to inhibit NAD degradation. NAD+ is essentially degraded (consumed) by poly (ADP-ribose) polymerase-1 (PARP-1) and CD38 (NAD hydrolase protein, converting NAD + into ADP-ribose or its cyclic counterpart). Inhibition of these degrading compounds increases NAD + levels (Navas and Carnero, 2021).

Intestinal microbiota contributes to the enhancement of NAD biosynthesis in host mammalian cells, an effect that synergizes with that of NAD-boosting supplements such as nicotinamide and nicotinamide riboside, and has been observed *in vitro* and *in vivo*. In fact, the increase of NAD levels in host organs following ingestion of precursors is highly dependent on healthy gut microbiota. Such an effect occurs because many organisms of the intestinal microbiota encode the pyrazinamidase/nicotinamidase PncA, a key component for alternative NAD salvage pathway in the host by engaging the deamidated biosynthesis pathway, the key-pathway of NAD biosynthesis in microorganisms (no homologs have been found in higher organisms). PncA enables the reconstruction of NAD using precursors such as nicotinic acid, nicotinamide riboside, and nicotinamide mononucleotide (Grozio et al., 2019; Yang et al., 2019).

More than 2000 PncA homologs, which have a isochorismate domain in common, have been detected in various bacterial species from different phyla (Shats et al., 2020). This indicating that NAD synthesis by the deamidated biosynthesis pathway is an extremely ancient evolutionary process, present in all biological kingdoms (Gazzaniga et al., 2009). Many members of the bacterial genera of mammalian microbiota harbor the PncA protein, including *Escherichia*, *Klebsiella*, *Salmonella*, *Fusobacterium*, *Enterococcus*, *Bifidobacterium*, *Lactobacillus*, *Clostridium* (but apparently not *Clostridioides*) or *Prevotella; w*ithin the phylum Bacteroidetes, PncA is rare among *Bacteroides* (Feng et al., 2021). In fact, if mice are treated with the PncA inhibitor pyrazinecarbonitrile, an analog of pyrazinamide (an anti-tuberculosis drug), the abundance of Clostridiales in the microbiota significantly decrease, showing its dependence on NAD deamidation synthesis. *E. coli* also is reduced by pyrazinecarbonitrile, demonstrating the importance of the deamidation NAD biosynthesis pathway in the proliferation of this potential pathogen, and possibly in Gamma-proteobacteria. Conversely, the anaerobic group of Bacteroidales, which lack PncA increased significantly (Feng et al., 2021), probably by ecological replacement of lost PncA harboring populations. Reduction in PncA bacterial populations in pyrazinecarbonitrile-treated mice significantly reduced a number of key-KEGG functions in the microbiota, including electron transfer, TCA cycle and respiration, amine and polyamine synthesis, and antibiotic resistance, all highly related to NAD + availability (Feng et al., 2021). The crosstalk between the host and its microbiome regarding NAD homeostasis has been experimentally studied. Germ-free mice were repopulated with wild *E. coli* harboring *pncA* or with the same strain with a non-functional *pncA* gene. Only in the first case was a marked increase in NAD metabolism detected in the mouse organs after gavage with nicotinamide (Shats et al., 2020). If cultures of wild *E. coli* (harboring PncA) are supplemented with nicotinamide, NAD + can be detected in the supernatant, and at higher levels if the gene *pncA* is hyper-expressed (Feng et al., 2021).

In the case of using activators of the NAD biosynthetic enzyme NAMPT, such as the aminopropyl carbazole P7C3-A20, the diversity (species richness) of the intestinal microbiota increases, meaning that an excess of NAD enriches minority (or slow growing) bacterial populations**.** In a study, certain genera were increased in proportions, such as *Akkermansia*, *Lactobacillus*, and Prevotellaceae, at the expenses of Enterobacteriaceae and *Parasutterella*, an important member of the core gut microbiota (Ju et al., 2019; Hua et al., 2021).

## Microbiota and Inhibitors of NAD Metabolism

Following the observations of Hasmann and Schemainda, 2003, NAD biosynthesis inhibitors have been developed to specifically de-energize fast-growing cancer cells. The rapid biogenesis of neoplastic cells require an increased amount of NAD, which is needed to protect tumor cells from the deleterious effects of superoxides, and to impede the degradation of dihydrofolate reductase, involved in accelerated DNA synthesis. Increased NAD levels favors cancer cell survival and dissemination (Pramono et al., 2020). NAD biosynthesis inhibitors are increasingly being considered as possible antineoplastic drugs (Roulston and Shore, 2016; Espindola-Netto et al., 2017; Chowdhry et al., 2019; Hong et al., 2020). In the salvage re-construction of NAD from the metabolites (e.g. nicotine) liberated by NAD-consuming enzymes (e.g., PARPs) the critical enzyme is NAMPT, which is hyper-expressed in tumor cells. NAMPT inhibitors strongly reduces the NAD and NADPH pool might then impair the growth of neoplastic cells (Galli et al., 2020; Pramono et al., 2020). Unfortunately NAMPT inhibitors not only depletes NAD from cancer cells, but also from many others, resulting in significant toxicity for the treated patient. Chronic intestinal inflammatory diseases also result in high NAD consumption, and NAMPT inhibitors are used as therapeutic agents (Gerner et al., 2018). However the microbiome itself might impair the therapeutic potential of these agents; if NAD is depleted by the use of NAD + synthesis inhibitors, the microbiota is able to counteract such an effect by expressing a series of proteins, mainly PncA (Feng et al., 2021). In fact, *E. coli*, and particularly microcinogenic strains (see below) are enriched in advanced colorectal tumors (Kohoutova et al., 2014). Microbiota composition and activity should therefore be taken into consideration when developing drugs that interfere NAD metabolism (Figure 1).

## NAD Metabolism and Antibiotic Persistent Bacteria

Persistence is a bacterial property associated with phenotypic (non-inheritable) resistance to antibiotics. in hosts treated with bactericidal drugs, minority populations of physiologically refractory survivors, the so-called “persisters” remain after treatment (Balaban et al., 2004; Bakkeren et al., 2020).

These bacteria do not grow or grow very slowly despite the presence of available nutrients. In general, bacteria’s susceptibility to death by bactericidal antibiotics is related to their replication rate (McCall et al., 2019). Little is known of the influence of persistent minority populations in the recovery of the gut microbiota following antibiotic exposure (Palleja et al., 2018). However, persisters in the gut are not-inactive and can promote the spread of antibiotic-resistance plasmids (Bakkeren et al., 2019).

Studies have recently discovered that a reduction in cellular NAD + levels favors the emergence of persistent cells; such reduction might influence the overall cellular metabolism, including redox homeostasis. Eventually, this might result from NAD + degradation by “toxins” belonging to “toxin-antitoxin” systems (composed of a stable toxin and a labile antitoxin) ensuring permanence of plasmids and chromosomal mobile genetic elements (Zhou et al., 2021). High-persistence gene A (*hipA*), is a type II toxin of a toxin-antitoxin system in *E. coli* K12. In the absence of antitoxin, this toxin strongly reduces growth rate and fosters formation of persister cells; likely by accumulation of tRNAGlu, activating the alarmone (p)ppGpp and inhibiting rRNA transcription; death might occur by depleting the intracellular NAD + pool (Korch and Hill, 2006; Zhang et al., 2020). The administration of NAD + boosting compounds might have an “awakening function” for persisters in the intestine facilitating microbiota recovery after antibiotic therapy. However, this administration might contribute to the increase in growth rate of persistent pathogens (e.g., *Salmonella*) making them more susceptible to antimicrobial agents and reducing carriership.

L-tryptophane, which enhances NAD + production, also reduces persister formation. In addition, the increase in proton motive force resulting from NAD production contribute to the increase in antibiotics uptake, as in the case of aminoglycosides, increasing their effectiveness (Rodriguez Cetina Biefer et al., 2017; Li et al., 2019). The acceleration of bacterial metabolism is probably a reason for decrease of persisters. The combination of serine (fostering the TCA cycle) with fluoroquinolones increases the NAD+/NADH ratio, the antimicrobial bactericidal effect and reduces the persisters’ emergence rate (Duan et al., 2016). Cysteine has a similar effect (Liu et al., 2020).

## NAD Metabolism and Microcin Peptide Antimicrobials

Processes designed to resuscitate and re-sensitize persister cells, and maintain NAD homeostasis, can be influenced or disturbed by natural antimicrobial agents of bacterial origin, such as peptide microcins, which are low-molecular-weight antimicrobial ribosomally synthesized substances produced mostly by Enterobacterales that modulate the structure of the host microbiome (Baquero and Moreno, 1984). Microcins are eco-active molecules that help maintain a healthy microbiota. Note that more than one-third of available *E. coli* genomes in databases contains microcin biosynthetic genes (Baquero et al., 2019; Rebuffat 2021). Notably, NAD + availability can be critical for maintaining enterobacterial populations and hence, microcin-producing strains. Indeed, studies with a NAD + -auxotrophic *E. coli* mutant have shown that supplementation with NAD + increases the *E. coli* growth rate (Zhou et al., 2011), which probably is the case with other microcin-producing Enterobacterales. The role of microcins in the gut ecology should be particularly relevant when Enterobacterales bloom in the inflamed gut (Sassone-Corsi et al., 2016), in some cases (as with microcin MccJ25) with an anti-inflammatory effect derived from the reduction in the NAD + availability. The following paragraphs discuss how microcin-expressing bacteria might modulate the NAD+/NADH ratio in the intestine.

The *E. coli* microcin MccB17 precursor is a 69-residue protein that is post-translationally modified by an enzyme complex, which convert 14 residues of the “core peptide” into 8 mono- and bis-heterocyclic moieties that confer antibiotic activity on mature microcin B17, the first known gyrase inhibitor of peptidic nature, and prototype of thiazole/oxazole-modified microcins (TOMMs).Four cysteine and four serine side chains undergo condensation with the carbonyl group of the preceding residue, followed by a critical step of NADH dehydrogenation that respectively results in the formation of four thiazole and four oxazole rings, and yields NAD+ (Li et al., 1996; Ghilarov et al., 2019). MccB17 is produced by *E. coli*, *Klebsiella pneumoniae* and probably other *Enterobacteriaceae*, given that it is plasmid-mediated (Baquero et al., 1978). The effect on DNA gyrase is linked to NADH oxidation using a pyruvate kinase, linking then the mechanisms of antimicrobial action to the NAD+/NADH pool. The same types of linear TOMMs are biosynthesized in a broad range of bacterial organisms, including *Streptococcus* (streptolysin) and *Listeria* (listeriolysin) (Rebuffat, 2021). Similar thiazole and oxazole rings were found in antitumor peptides of microbial and marine origin, including cyanobacteria symbionts, and in bacterial siderophores (Schmidt et al., 2005; Melby et al., 2011), supporting that this types of compounds are broadly distributed.

The *E. coli* microcin MccJ25 has a peculiar post-translational structure consisting of a lasso-like ring of 8 amino acids and a C-terminal tail of 13 amino acids folded upon itself which passes through the ring. Inhibition of the respiratory chain is one of the causes for the MccJ25 toxic effect on *E. coli* and *Salmonella* Newport. MccJ25 is an inhibitor of RNA polymerase and increased superoxide production, inhibiting NADH dehydrogenase, and reducing the availability of NAD + through the flow of electrons to terminal oxidases (with oxygen consumption) (Bellomio et al., 2007; Galván et al., 2019). Such effect might contribute explaining the beneficial effect of MccJ25 in inflammatory gut processes (and perhaps in cancer?) (Yu et al., 2020; Shang et al., 2021). However, to what extent the activity of this microcin and others with similar mechanism of action may serve to keep (or modify) the NAD homeostasis in the gut remains to be established, probably been related to the abundance of microcinogenic organisms in the intestine.

The *E. coli* microcin MccC7 is a nucleotide-associated ribosomally-produced heptapeptide. The bactericidal effect of MccC7 is correlated with the inhibition of respiration chain dehydrogenases, and the massive inhibition of enzyme activity (Ran et al., 2017). MccC7 induces bacterial persistence, by inducing, very much as HipA (p)ppGpp synthesis. As microcin MccC7 is excreted, it influences the emergence of persisters in surrounding cells (Piskunova et al., 2017). Cells exposed to MccC7 probably have a reduced NAD+/NADH rate, and NAD + boosting is expected to reduce the effect of this microcin.

Microcins, natural compounds, can be considered in the design of future antibacterial agents targeting NADH dehydrogenases (Collin and Maxwell, 2019).

## Discussion. Microbiota Modulation and Drugs Acting on NAD Metabolism: Clinical Considerations

Based on the above, it could be assumed that the administration of oral NAD-boosting supplements might modulate the intestinal microbiota favoring the growth of PncA organisms, which could result in a beneficial effect for hosts being treated, for instance, for neurological disorders. However, NAD-boosting supplementation might increase the abundance of potential pathogens frequently causing bacteremia in elderly patients, such as *E. coli*, *K. pneumoniae*, *Enterococcus faecium, Enterococcus faecalis* or *Salmonella* Typhimurium.

Fortunately, the antibiotics bactericidal action can be influenced by NAD metabolism; however, this is a poorly explored field of research. In *E. coli*, NAD + fosters metabolic overflow, accelerated respiration and reactive oxygen species formation, which contribute to the antibiotic’s bactericidal effect (Dwyer et al., 2014; Lobritz et al., 2015; Baquero and Levin, 2021). Stimulating proton motive force, increasing antibiotic uptake, and activating the tricarboxylic acid cycle facilitates bacterial killing (Banerjee et al., 2017).

Most patients with neurological diseases treated with NAD metabolism enhancer drugs are of advanced age, in whom the microbiota is altered, resulting in a possible decrease in metabolic capacity of carbohydrate degradation, attributed to the reduction in *Bifidobacterium* (An et al., 2021)*,* due to NAD limitation. If certain pathogens increase in abundance, NAD boosting might favor beneficial organisms, particularly in combination with probiotics, prebiotics, or fecal transplantation.

Antibiotics are frequently administered to the elderly, due to the repeated infective processes (Tedim et al., 2015; Rodríguez et al., 2021), and the beneficial boosting effect of microbiota on the action of NAD enhancer drugs can be reduced by antibiotic uptake, particularly of drugs with high concentrations in the gut (oral aminoglycosides and polymyxins, or bile-excreted antimicrobials, as ceftriaxone) which should be avoided during NAD-enhancers therapy.

Unfortunately, most patients with frequent neurological diseases are of an age in which the proportion of neoplastic events also increases. If the intestinal microbiota increases the effect of NAD boosting drugs, their concomitant use with antimicrobial agents should be prescribed only in patients where the presence of neoplastic disease can be ruled out. Bacteria are frequently associated with tumors (Xu et al., 2020; Dumont-Leblond et al., 2021); for instance, the bronchial environments of lung tumors bronchial is enriched in *Streptococcus* harboring PncA nicotine amidase (Bello et al., 2021). The activity of this bacterial protein might protect neoplastic tissues from NAD-targeting anti-cancer drugs by supplying nicotinic acid to the tumor cells, feeding the deamidated biosynthesis pathway to fulfill their high demand for NAD. If it were the case, antibiotics could slow the progression of tumoral tissues.

New possibilities for employing NAD + boosting agents as modulators of the intestinal microbiota should be considered for fostering repopulation of microbiota after decontaminating antibiotic therapy, for instance in graft-versus-host disease (e.g., after allogeneic hematopoietic cell transplantation); for “awakening” phenotypically resistant (persistent) populations making them more susceptible to antimicrobial therapy with the effect of shortening carriership, and for fecal transplantation procedures to enhance the implantation of the administered microbiota, presumably including therapy for *Clostridioides difficile* decontamination.
